# A new species of *Aciconula* (Amphipoda, Senticauda, Caprellidae) from Sultan Iskandar Marine Park, Malaysia

**DOI:** 10.3897/zookeys.859.33284

**Published:** 2019-07-02

**Authors:** Jacqueline H.C. Lim, B. Abdul Rahim Azman, B.H. Ross Othman

**Affiliations:** 1 School of Environmental & Natural Resource Sciences, Faculty of Science and Technology, Universiti Kebangsaan Malaysia, 43600 UKM Bangi, Selangor, Malaysia; 2 Marine Ecosystem Research Centre (EKOMAR), Faculty of Science and Technology, Universiti Kebangsaan Malaysia, 43600 UKM Bangi, Selangor, Malaysia; 3 School of Marine and Environmental Sciences, Universiti Malaysia Terengganu, 21030 Kuala Nerus, Terengganu, Malaysia

**Keywords:** *Aciconulatinggiensis* sp. nov., caprellid, new species, taxonomy, South China Sea

## Abstract

A new species of caprellid, *Aciconulatinggiensis* (Amphipoda, Senticaudata, Caprellidae) was discovered from Pulau Tinggi, Sultan Iskandar Marine Park (SIMP), South China Sea, Malaysia. The new Malaysian species can be distinguished from the other *Aciconula* species by the combination of the following characters: 1. the presence of a very small suture between head and pereonite 1; 2. antenna 1 flagellum with 4 articles; 3. inner lobe of lower lip unilobed; 4. gnathopod 2 palm of propodus with a large proximal projection (stretching from the proximal margin of the palm to nearly mid-way of palm); 5. pereopods 3–4 with 2 articles (article 1 subrectangular, article 2 conical or tapering at the tip with 1 plumose seta and 2 normal setae) and; 6. pereopod 5 covered with relatively dense and long setae. An updated identification key for the five known species in the genus, including information on the respective geographical distribution and habitat, is presented.

## Introduction

The amphipod genus *Aciconula* was established by Mayer (1903) with *A.miranda* Mayer, 1903 as its type species. However, the exact diagnostic characteristics of this genus were unclear because the types described by Mayer (1903) for this genus were both females (that were collected from three different localities: Singapore, Malaysia and Koh Krau, Thailand) and only figures of the whole body, pereopod 3, pereopod 4, pereopod 5, mandibular palp article 3 and maxilliped were drawn. Mayer (1912) then described a male but the abdomen and mouthparts were also not included. Nevertheless, general morphology of the male specimen agrees well with the type and the rest of the appendages such as pereopods 3 to 7 are also similar to the female. The abdomen was also left out. Following that, Arimoto (1976) referred to Utinomi’s (1969) description based on specimens collected from Kii Peninsula, Japan and revised its generic diagnosis. Subsequently, three more species of *Aciconula* were reported; *Aciconulaacanthosoma* Chess, 1989 from southern California; *A.australiensis* Guerra-García, 2004 from Western Australia and most recently *A.tridentata* Guedes-Silva & Souza-Filho, 2013 described from Pernambuco, Brazil.

**Figure 1.**
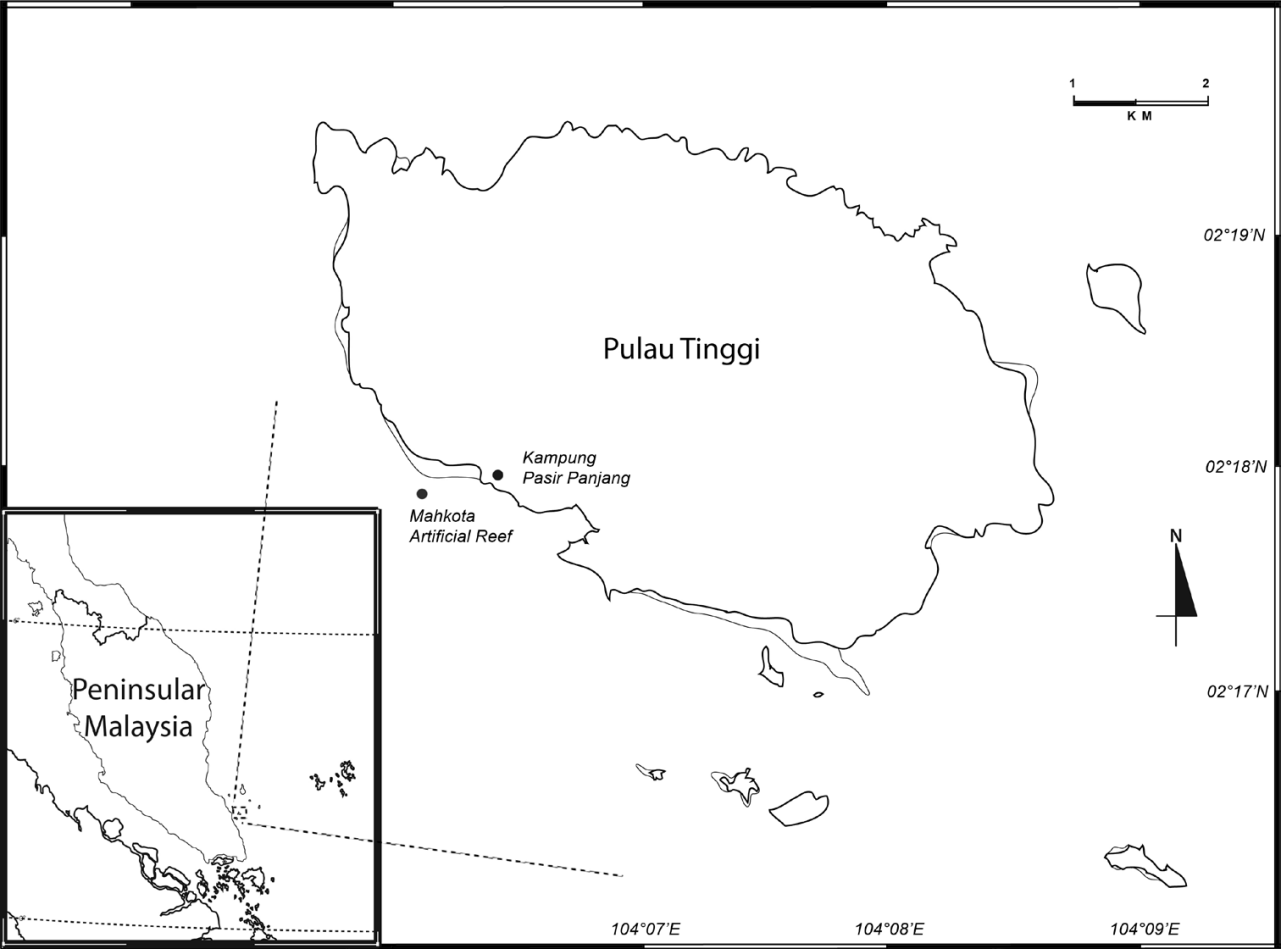
Pulau Tinggi of Sultan Iskandar Marine Park (SIMP), Malaysia.

The Sultan Iskandar Marine Park (SIMP) is one of Malaysia’s marine protected areas located 15–65 km from Mersing, off the north-east coast of the Johor State, Malaysia in the South China Sea. This body of water covering an area of about 8000 hectares holds one of the most diverse marine ecosystems on the east coast of Peninsular Malaysia, ranging from sandy shores, coral reefs, mangroves, estuaries, mudflats to seagrass and open water habitats (see Harborne et al. 2000; Japar Sidik and Muta Harah 2003; Maritime Institute of Malaysia 2006; Japar Sidik et al. 2006; Azman et al. 2008; Japar Sidik and Muta Harah 2011; Lim et al. 2015). The SIMP (Fig. 1) consists of 13 main islands namely Pulau Harimau, Pulau Mensirip, Pulau Goal, Pulau Besar, Pulau Tengah, Pulau Hujung, Pulau Rawa, Pulau Tinggi, Pulau Mentinggi, Pulau Sibu, Pulau Sibu Hujung, Pulau Pemanggil and Pulau Aur. This paper continues the previous significant contributions on the biodiversity of SIMP and its vicinity including Othman and Azman (2007), Lim et al. (2010), Gan et al. (2010), Azman and Melvin (2011), Lim et al. (2012), Azman and Othman (2013), Chew et al. (2014), Tan et al. (2014, 2015), Lim et al. (2015), Chew et al. (2016)Lim et al. (2017) and Tan and Azman (2017, 2018) on marine crustaceans. The present paper also deals with the detailed description of this new species; an updated identification key to all the known *Aciconula* species is also given.

## Material and methods

### Sampling

The caprellids examined in this study were collected from an artificial reef of Kampung Pasir Panjang, Pulau Tinggi, Sultan Iskandar Marine Park (SIMP) at 9–11 m water depth (Fig. 1). Collections were made by SCUBA; hosts (stinging hydroids) together with attached caprellids were put into fine mesh bags. Specimens used for morphological descriptions were preserved in 4% formaldehyde before examination.

### Laboratory procedures

Appendages were dissected from the right side of the specimens and stored in several semi-permanent slides mounted in glycerol and then drawn under an optical microscope (Olympus BX43) and a stereomicroscope with a camera lucida. The drawings were digitized on Adobe Illustrator CS3 using the methods described in Coleman (2003). All materials are deposited at the South China Sea Research and Repository Centre, Institute of Oceanography and Environment, Universiti Malaysia Terengganu, 21030 Kuala Nerus, Terengganu, Malaysia (UMT Crus). The following abbreviations are used in the figures: **A**, antenna; **ABD(L)**, abdomen lateral view; **ABD(V)**, abdomen ventral view; **G**, gnathopod; LL, lower lip; **MD**, mandible; **MX**, maxilla; **MXP**, maxilliped; **P**, pereopod; **UL**, upper lip; **R**, right; **L**, left; ♂, male; ♀, female.

## Systematics

### Order AMPHIPODA Latreille, 1816

#### Suborder COROPHIIDEA Leach, 1814

##### Family CAPRELLIDAE Leach, 1814

###### Genus *Aciconula* Mayer, 1903

####### Aciconula tinggiensis sp. nov.

Taxon classificationAnimaliaAmphipodaCaprellidae

http://zoobank.org/DEE62918-DA9C-4090-9245-872B7D982DBD

Figs 2 , 3 , 4 , 5

######## Etymology.

Named after the type locality, Pulau Tinggi in SIMP, Malaysia.

######## Material examined.

Holotype: male, 2.2 mm, UMT Crus 01003, Mahkota artificial reef Pulau Tinggi, SIMP, Johor, 02°17.637'N, 104°05.817'E, SCUBA diving, 9 June 2009, 12.31 PM, depth 10.7 m, coll. Azman, B.A.R., Gan, S.Y., Lim, J.H.C., Chew, M.W.H. & Shamsul, B.

Paratypes: 1 female, UMT Crus 01004 (Fig. 4); 2 males, 1 female, UMT Crus 01005; 2 males, 2 females, UMT Crus 01006; 2 males, 2 females, 1 juvenile, UMT Crus 01007; same station data.

######## Type locality.

Mahkota artificial reef, Pulau Tinggi, SIMP, Malaysia.

**Figure 2.**
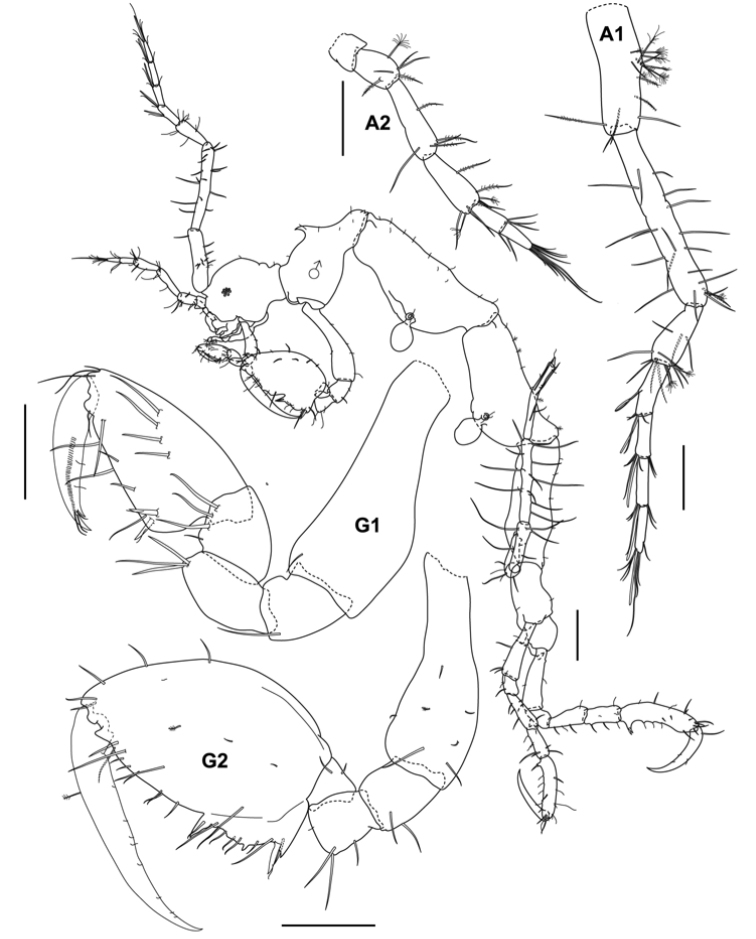
*Aciconulatinggiensis* sp. nov., male holotype, 2.2 mm, UMT Crus 01003, Mahkota artificial reef, Pulau Tinggi, SIMP. Scales bars: 0.1 mm (A1, A2, G2); 0.05 mm (G1); 0.2 mm (whole body).

######## Description.

[Based mostly on holotype (UMT Crus 01003), 2.2 mm, supplemented by paratype (UMT Crus 01004), 1.8 mm for female and (UMT Crus 01005) for lower lip and maxilliped]

Adult male. Body length, 2.2 mm. UMT Crus 01003. Head/pereonite 1 without dorsal projection. Head length 0.2 mm; pereonite 1, 0.07 mm; head and pereonite 1 partially fused (suture clear); pereonite 2, 0.34 mm with an acute mid-dorsal projection; pereonite 3 longest, 0.53 mm; pereonite 4, 0.44 mm; pereonite 5, 0.41 mm, subequal in length to pereonite 4; pereonite 6, 0.16 mm; pereonite 7 short, 0.12 mm. Eye small. Antenna 1 about 0.4× body length; peduncular article 1 with tuft of plumose setae; peduncular article 2 longest; peduncular article 3 lobed at posterodistal margin; flagellum approximately half of peduncular length with 4 articles, proximal article composed of 2 articles. Antenna 2 about 0.4× the length of antenna 1; peduncular articles lobus; flagellum 2-articulate.

Lower lip outer lobes with pair of ducts; inner lobes unilobed. Mandible left incisor with 6 teeth; lacinia mobilis plate like and serrated distally; accessory setal row with 3 setae; mandibular molar present without robust teeth; palp 3-articulate with distal article comprising a row of 5 teeth and setal formula of 1-3-1, second article of palp without seta on inner distal margin; right incisor with 5 teeth; lacinia mobilis with 7 teeth; accessory setal row with 2 setae; palp 3-articulate with distal article comprising a row of 5 teeth and setal formula of 1-3-1, second article of palp with one seta on inner distal margin. Maxilla 1 outer plate with 5 cuspidate and denticulate spines (robust apical setal-teeth); palp article 2 long, 4× length of article 1 with 3 setae apically. Maxilla 2 inner plate with 4 short and long setae distally; outer plate 1.3× length of inner plate with 5 slender setae apically. Maxilliped inner plate small, with one short and one long apical setae; outer plate about 2.5× inner plate with 3 setae at distal margin; palp 4-articulate, scarcely setose, article 2 with 1 seta at inner distal margin, article 3 with 5 distal setae; article 4 tapering to a tip with 2 setae distally and 1 seta at outer proximal margin.

Pereon. Gnathopod 1 basis longer than ischium, merus and carpus combined; propodus subtriangular, longer than wide, scarcely setose, palm with a pair of grasping spines; dactylus falcate, provided with fine setae along lateral margin, tip of dactylus bifid. Gnathopod 2 begins ¼-way along anterior margin of pereonite 2; basis about 0.7 × pereonite 2; ischium and merus subquadrate; carpus triangular; propodus 1.6 × as long as wide, 1.3× length of basis, palm with large proximal projection (stretching from proximal margin of palm to nearly mid-way of palm), provided with one robust grasping spine proximally, a small triangular projection medially and ending with a triangular projection provided with 1 seta, distal margin of palm with 1 triangular projection; dactylus falcate, fitting on palm.

**Figure 3.**
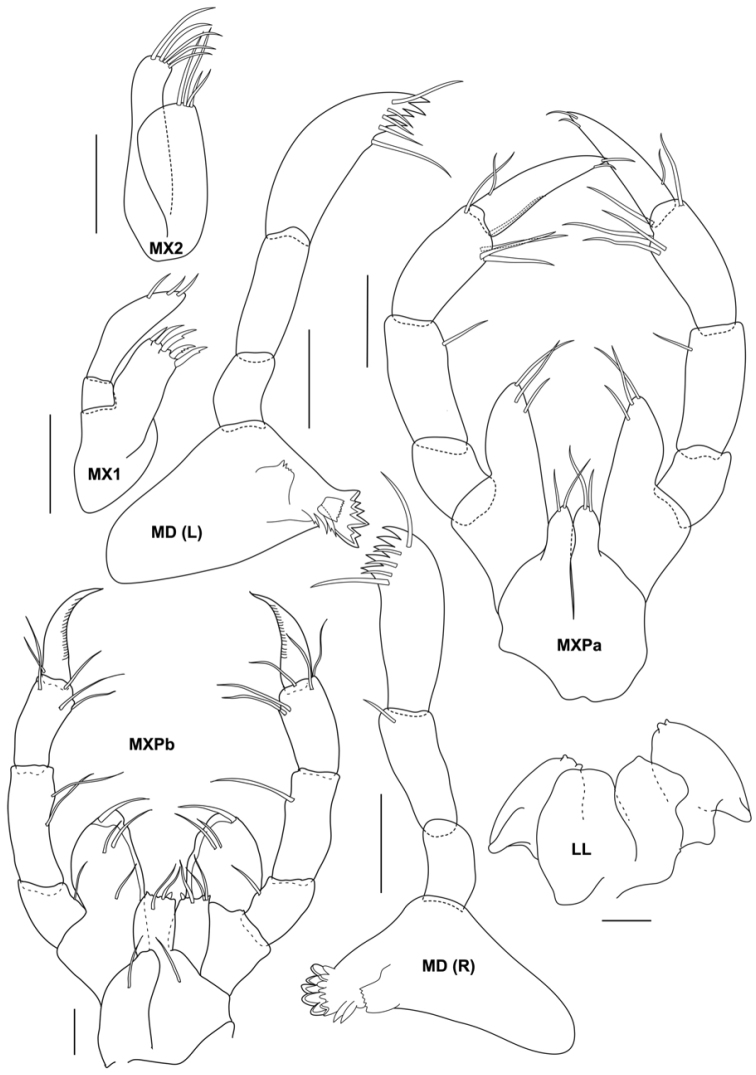
*Aciconulatinggiensis* sp. nov., male holotype, 2.2 mm, UMT Crus 01003, Mahkota artificial reef, Pulau Tinggi, SIMP. Lower lip (LL) and MXPb from male paratype, UMT Crus 01005. Scale bars: 0.025 mm

Gill 3 length 0.2× corresponding pereonite, oval. Pereopod 3 reduced, about 0.5× gill length, 2-articulate, second article with one plumose seta and two simple setae apically. Gill 4 slightly larger than gill 3, 0.3 × corresponding pereonite, oval. Pereopod 4 reduced, about 0.5 × gill length, 2-articulate, second article of pereopod 4 more slender than article 2 of pereopod 3 with one plumose seta and two simple setae apically. Pereopod 5, 6-articulate, curved upwards anterodorsally and extending past pereonite 4, setose entire margin comprising short and very long setae, carpus and propodus subequal in length, dactylus reduced to a small cone with one plumose seta apically. Pereopod 6 propodus with a pair of grasping spines proximally, dactylus falcate with one plumose seta on anterior margin at proximal region. Pereopod 7 similar with pereopod 6 but more robust than pereopod 6.

Pleon. Uropod 1 vestigial with 4 setae; Uropod 2 vestigial with 2 setae distally and one facial seta on inner margin. Telson with one seta apically.

Adult female. Body length, 1.8 mm. UMT Crus 01004. Head length 0.2 mm; pereonite 1, 0.04 mm; head/pereonite 1 without dorsal projection; pereonite 2, 0.29 mm with rounded mid-dorsal projection; pereonite 3, 0.39 mm; pereonite 4, 0.32 mm with acute dorsodistal projection; pereonite 5, 0.38 mm, subequal in length to pereonite 3; pereonite 6, 0.13 mm; pereonite 7 short, 0.08 mm. Eye small. Antenna 1 about 0.4 × body length; peduncular article 1 with tuft of setae; peduncular article 2 longest; peduncular article 3-lobed at posterodistal margin; flagellum approximately 1.8 × peduncular length with 4 articles. Antenna 2 about 0.4 × the length of antenna 1; peduncular articles lobus; flagellum 2-articulate.

Mouthparts of the female are similar to those of male (refer to male mouthparts).

Pereon. Gnathopod 2 basis begins ¼-way along anterior margin of pereonite 2; basis about 0.7 × pereonite 2; ischium and merus subquadrate; carpus subtriangular; propodus 2.4 × as long as wide, 1.2 × length of basis, palm without large proximal projection, provided with one robust grasping spine distally; dactylus falcate, fitting on palm. Gill 3 length 0.3 × corresponding pereonite, oval. Pereopod 3 reduced, about 0.5 × gill length, 2-articulate, similar with the male, second article with one plumose setae and two simple setae apically. Gill 4 subequal with gill 3, 0.4 × corresponding pereonite, oval. Pereopod 4 reduced, about 0.4 × gill length, 2-articulate, subequal with pereopod 3 with one plumose setae and two simple setae apically. Oostegites on pereonite 3 and 4 with setae. Pereopod 5, 6-articulate, curved upwards anterodorsally and extending past pereonite 4, more slender than male pereopod 5, setose entire margin comprising short and very long setae, propodus longest, dactylus reduced to a small cone with one plumose seta apically.

Pleon. Uropod 1 vestigial with 1 simple setae; Uropod 2 vestigial with 1 setae distally. Telson with one plumose seta apically.

**Figure 4.**
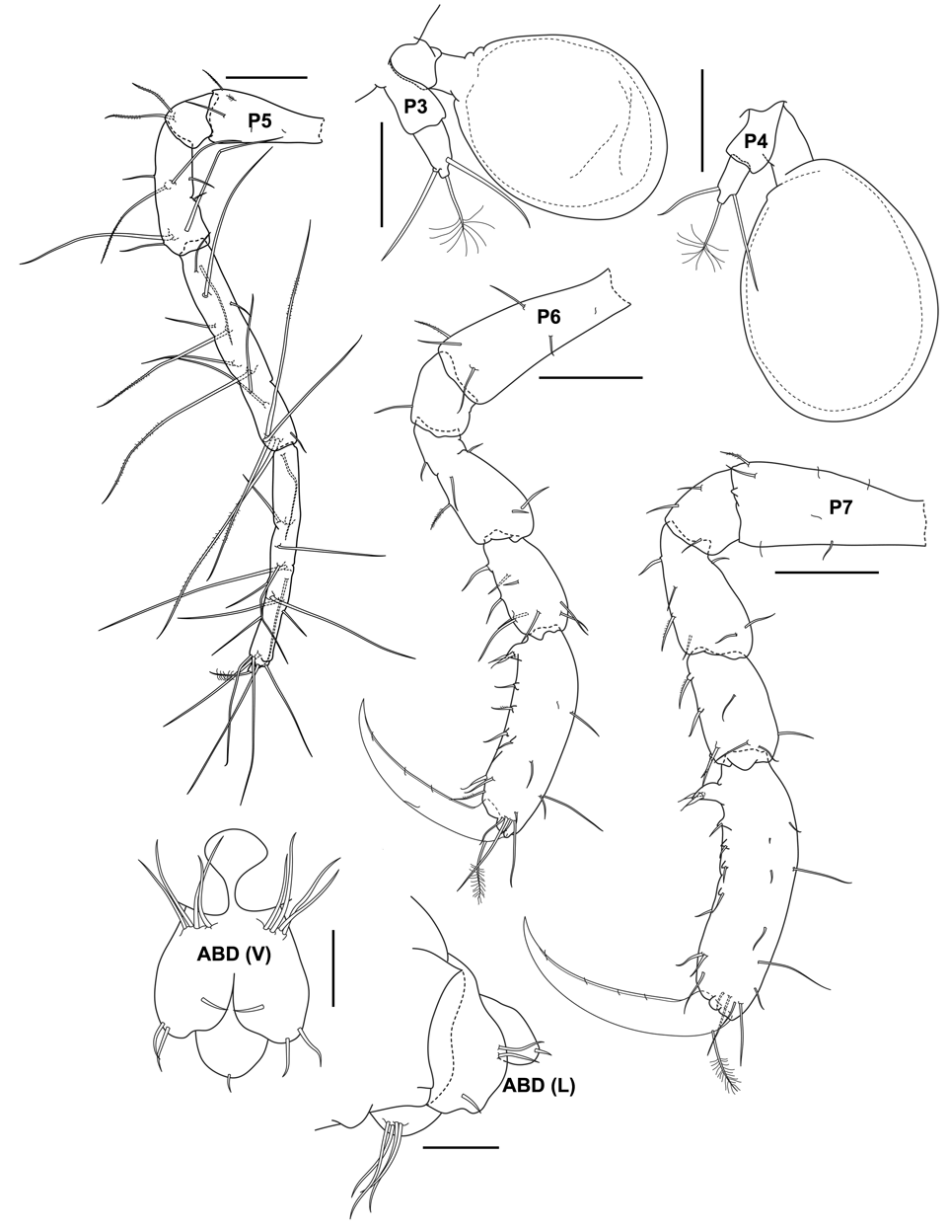
*Aciconulatinggiensis* sp. nov., male holotype, 2.2 mm, UMT Crus 01003, Mahkota artificial reef, Pulau Tinggi, SIMP. Scale bars: 0.025 mm (ABD); 0.05 mm (P3, P4); 0.1 mm (P5, P6, P7).

######## Remarks.

Considering the four reported species from the genus *Aciconula*, *A.tinggiensis* sp. nov. is most similar to *A.australiensis* in terms of antenna 1 and 2, gnathopod 1, mouthparts (maxilliped and maxillas) and abdomen. Pereopods 3 and 4 of the male are also similar except for the presence of a seta on article 1 of *A.australiensis*. The Malaysian specimen differs from the Australian counterpart in terms of the absence of 1) a head projection (present in *A.australiensis*); 2) inner lobe of lower lip unilobed (*A.australiensis* bilobed); 3) gnathopod 2 propodus proximal projection shallow and wide, about 1/2 of palm (*A.australiensis* more pronounced, about 1/3 of palm); 4) pereopod 3 of female similar to the male with only 2 articles while *A.australiensis* shows sexual dimorphism with 3 articles; 5) longer (articles 4 and 5 about 2 × longer) pereopod 5, 0.43 × body length, terminal article with one plumose seta and generally more setose (*A.australiensis* only 0.23 × body length; terminal articles with one normal seta); 6) mandibles with setal formula of 1-3-1 (setal formula 1-4-1 in *A.australiensis*).

*Aciconulaacanthosoma* Chess, 1989 clearly differs from the Malaysian specimen firstly by its numerous dorsal projections throughout its body, maxilliped inner lobes more robust and wide, terminally with one tooth and three setae (slender with two normal setae in *A.tinggiensis* sp. nov.), maxilla 2 with very short terminal setae and mandibles with large, well-developed molar and palp with setal formula of 1-6-1. Apart from its body armature and mouthparts, it also varies in terms of appendages such as gnathopod 2 (basis with a distolateral projection, palmar margin of propodus with a proximal projection followed by a strong spine and a deep sinus), pereopod 5 with short and fine dense setae (setae longer and less extensive in *A.tinggiensis* sp. nov.) , and abdomen with one pair of well-developed, 1-articulate abdominal appendage. The species from southern California is also much larger than the present specimen (3.3 × longer) and the other two existing species from the Indo-Pacific. In spite of this, *A.acanthosoma* does have a few similarities in pereopods 3 and 4 with 2 articles and article 2 conical (except for the 3 terminal plumose setae) and pereopods 6 and 7 with 7 articles and grasping structure on article 6. There is more dissimilarity in these two species than similarities. According to Guerra-García (2004), *A.acanthosoma* could be placed in a new genus based on the abdominal appendages and several mouthparts but presently there is only one genus, which exhibits the soft and flexible character of pereopod 5, therefore it is retained in the genus *Aciconula*.

The female specimen of *A.tinggiensis* sp. nov. was primarily used to compare with Mayer’s (1903) type and Mayer’s (1912) account of *A.miranda* because he only provided more detailed description and figures of females compared to males. Females of *A.tinggiensis* sp. nov. are similar to *A.miranda* Mayer, 1903 based on a single dorsal projection on pereonite 2, antenna 1 flagellum with 4 articles, pereopod 4 with 2 articles (similar in shape and terminally with one plumose seta), and its gill shape. However, the present species (female) differs from *A.miranda* Mayer, 1903 in its pereopod 5 which has longer but less dense setae (more densely setose in *A.miranda*); pereopod 3 with 2 articles (3 articles in *A.miranda*); and pereopod 3 article 2 is conical and short (article 2 is subcylindrical with several seta marginally). Mouthparts of *A.tinggiensis* sp. nov. for males and females are similar therefore only the male mouthparts are used for comparison with Mayer’s (1903) description and figures. *Aciconulatinggiensis* sp. nov. differs from *A.miranda* in terms of the setal formula of the mandibular palp 1-3-1 (setal formula 1-7-1 in *A.miranda*) and maxilliped outer plate with only 3 distal setae (maxilliped entire inner margin lined with setae in *A.miranda*).

*Aciconulatridentata* Guedes-Silva & Souza-Filho, 2013 reported from Brazil, is similar to the present species in the: 1) presence of a small sharp median forward projection of the head; 2) pereopods 3 and 4 of male with two-articles, and absence of abdominal appendages but differs in the length of the outer plate of maxilliped (longer in *Aciconulatinggiensis* sp. nov. reaching the mid-length of palp article 2), the sculpturing on the palm of male gnathopod 2, (with a 3-dentate projection, followed by a large excavation leading to a projection with two sharp processes in *A.tridentata*) and number or articles in female pereopods 3 and 4 (pereopod 3 4-articulate and pereopod 4 3-articulate in *A.tridentata*)

In conclusion, *Aciconulatinggiensis* sp. nov. described here is recognized as distinct from the four existing species of this genus based on these combination of characters; 1) a very small suture between head and pereonite 1; 2) antenna 1 flagellum with 4 articles, its setal formula of 1-3-1; 3) unilobed inner lobe of lower lip with pair of ducts on outer lobe; 4) gnathopod 2 palm of propodus with a large proximal projection, (stretching from the proximal margin of the palm to nearly mid-way of palm) provided with one robust grasping spine proximally, a small triangular projection medially and ending with a triangular projection provided with 1 seta; 5) pereopods 3 and 4 with 2 articles (article 1 subrectangular, article 2 conical or tapering at the tip with 1 plumose seta and 2 normal setae); 6) pereopod 5 covered with relatively dense and long setae; and 7) abdomen region with penes situated medially, uropod 2 degenerated into 4 setae, uropod 2 degenerated into 1 seta medially and 2 setae distally.

######## Habitat.

The specimens have been found from 10–12 meters deep, living on stinging hyroids.

######## Distribution.

Currently only known from Pulau Tinggi, Johor, Malaysia.

**Figure 5.**
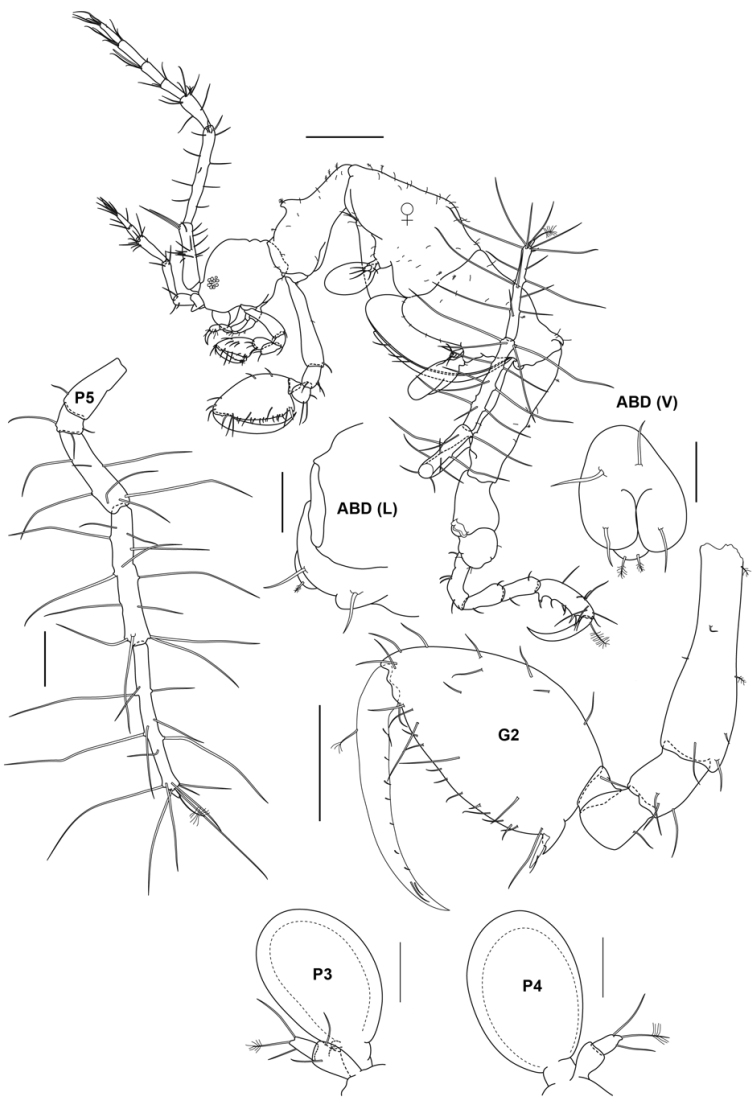
*Aciconulatinggiensis* sp. nov., female paratype, 1.8 mm, UMT Crus 01004, Mahkota artificial reef, Pulau Tinggi, SIMP. Scale bars: 0.025 mm (ABD); 0.1 mm (G2, P5); 0.05 mm (P3, P4); 0.2 mm (whole body).

### Key to the species of the genus *Aciconula* Mayer, 1903

**Table:** 

1	Body dorsally strongly spinose, abdominal appendages present	***A.acanthosoma* Chess, 1989**
–	Body dorsally not spinose, abdominal appendages absent	**2**
2	Head lacking a small sharp median forward projection	***A.miranda* Mayer, 1903**
–	Head with a small sharp median forward projection	**3**
3	Antenna 1 article 1 bearing a setose hump proximally	***A.australiensis* Guerra-Garcia, 2004**
–	Antenna 1 article 1 lacking setose hump proximally	**4**
4	Gnathopod 2 male first half of propodus palm bearing a 3-dentate followed by a large excavation	***A.tridentata* Guedes-Silva & Souza-Filho, 2013**
–	Gnathopod 2 male first half of propodus palm without dentation	***A.tinggiensis* sp. nov.**

## Supplementary Material

XML Treatment for Aciconula tinggiensis
